# Development of a Direct and Rapid Detection Method for Viable but Non-culturable State of *Pediococcus acidilactici*

**DOI:** 10.3389/fmicb.2021.687691

**Published:** 2021-07-02

**Authors:** Yu Guan, Kan Wang, Yang Zeng, Yanrui Ye, Ling Chen, Tengyi Huang

**Affiliations:** ^1^Department of Urology, Beijing Friendship Hospital, Capital Medical University, Beijing, China; ^2^Center for Translational Medicine, The Second Affiliated Hospital of Shantou University Medical College, Shantou, China; ^3^Shantou University Medical College, Shantou, China; ^4^School of Biological Science and Engineering, South China University of Technology, Guangzhou, China; ^5^School of Food Science and Engineering, Guangdong Province Key Laboratory for Green Processing of Natural Products and Product Safety, South China University of Technology, Guangzhou, China; ^6^Department of Laboratory Medicine, The Second Affiliated Hospital of Shantou University Medical College, Shantou, China

**Keywords:** *Pediococcus acidilactici*, viable but non-culturable, artificially contaminated food, PMA-CPA, rapid detection, safety control

## Abstract

*Pediococcus acidilactici* may significantly reduce the pH-value, and thus has different influence, including serving as a probiotic in human microbiota but a spoilage in human food as it could change the flavor. *Pediococcus acidilactici* is also capable of entering into the viable but non-culturable (VBNC) state causing false negative results of standard culture-based detection method. Thus, development of detection method for VBNC state *P. acidilactici* is of great significance. In this study, propidium monoazide (PMA) combined with cross priming amplification (CPA) was developed to detect the VBNC cells of *P. acidilactici* and applied on the detection in different systems. With detection limit of 10^4^ cells/ml, high sensitivity, and 100% specificity, PMA-CPA can successfully detect VBNC cells of *P. acidilactici* and be applied in with high robustness.

## Introduction

*Pediococcus acidilactici* may significantly reduce the pH-value, and thus has different influence, including serving as a probiotic in human microbiota but a spoilage in human food. Most commonly, *P. acidilactici* has been considered to be a probiotic bacteria for human beings, and also a typical type of lactic acid bacteria (LAB), which have been well-documented to be widely existing in human microbiota. Such probiotic bacteria have been well-studied to significantly aid in the microbiota balance, and once altered, various types of human diseases could occur. One important example is the correlation between human microbiota and urinary tract infection, in which microbiota in a few different sites, including intestine and vagina, has been found to play a role in the occurrence of urinary tract infection (Foxman, 2010; Whiteside et al., 2015; Stapleton, 2017; Paalanne et al., 2018; Magruder et al., 2020). Therefore, probiotic LAB is an important type of human probiotic and significantly aids in the prevention of urinary tract infection. In addition, accurate detection of probiotic commensal has significantly raised the public interest as this serves as an important indicator. However, a major concern still remains, as a large proportion of such probiotic commensal are hard to culture, which has further raised the aim of this study. As a commonly existing human bacteria, *P. acidilactici* is also one of LAB that are frequently used due to their capacity to produce bacteriocin to inhibit the growth of other spoilage bacteria (Salminen et al., 2004; Zhong et al., 2013). However, with improper processing, *P. acidilactici* produces acids including lactic acid, malic acid, citric acid, propionic acid, acetic acid, and short-chain fatty acids through fermentation, resulting in the significant decrease of pH-value, and the change in morphology and flavor (Olaoye et al., 2008; Xu et al., 2009). In consequence, the shelf life of the food would be shorter and the deterioration of the food occurs. Furthermore, it has been proved that *P. acidilactici* is able to enter into the viable but non-culturable (VBNC) state, which causes the false negative detection by the standard culture-based detection methods (Fakruddin et al., 2013; Ramamurthy et al., 2014; Xie et al., 2017a,b; Xu et al., 2017a,b; Li et al., 2020). Thus, accurate detection of *P. acidilactici* cells in the VBNC state is in need (Ding et al., 2017; Truchado et al., 2020).

Nowadays, the detection of the VBNC state is currently based on molecular detection techniques and the fluorescence microscopy with dying kit (Xu et al., 2011a,b,c; Zhao et al., 2018a,b; Dong et al., 2020). In the past few years, PCR based methodologies have been developed for rapid detection of viable bacterial cells, in combination with use of different substances including propidium monoazide (PMA) (Zhong et al., 2016; Bao et al., 2017a,b,c; Jia et al., 2018; Liu et al., 2018b,c; Zhong and Zhao, 2018). However, PCR based methodologies require strict temperature change process and determination step, such as gel electrophoresis or hybridization, which significantly reduces rapidity and simplicity in operation of such methods (Miao et al., 2016, 2017a,b,c; Zhao et al., 2020). In addition, the PMA-PCR has a lower sensitivity compared to the novel isothermal amplification methods with complex procedure and higher cost (Xu et al., 2008a,b; Liu et al., 2019).

Cross priming amplification (CPA) was developed to detect the target DNA with exponential amplification (Xu et al., 2007, 2010; Bai et al., 2015). Cross priming amplification can be completed under constant temperature with the advantages of simplicity, rapidity, high sensitivity, and cost-efficiency (Liu et al., 2015, 2017; Zheng et al., 2020). The expensive heating machine can be replaced by the simple water bath or other heating block. The whole amplification procedure can be completed within 1 h by one pair of primer, which spans three distinct sequences of a target gene (Lin et al., 2016; Zhang et al., 2019). The products of CPA can be measured based on the turbidity, electrophoresis of amplicons, and DNA-specific fluorescent reaction in tubes, such as SYBR Green-I (Xu et al., 2012b,c, 2016a,b,c; Lin et al., 2017).

In a previous study (Li et al., 2020), we had studied the key conditions of VBNC formation of *P. acidilactici*, and then based on the key conditions, we had discovered the reduction of VBNC formation. However, a major concern still remains, as rapid detection of *P. acidilactici* is highly required, especially direct detection and real-time surveillance from food products. Consequently, in this study, first we developed a rapid, sensitive, and specific detection assay on *P. acidilactici* based on CPA methodology. Then, we had further developed a PMA-CPA assay to directly identify the VBNC cells of *P. acidilactici*. Last, we had applied the established methodology on three different types of food products.

## Materials and Methods

### Strains and Culturing

A total of 20 bacterial strains, which are common foodborne pathogens and spoilage bacteria, were tested in this study, including a *P. acidilactici* strain, and 19 non-target bacteria (Table 2), including *Escherichia coli, Salmonella, Staphylococcus aureus, Listeria monocytogenes, Vibrio parahaemolyticus, Lactobacillus casei, Lactobacillus acetotolerans, Lactobacillus plantarum*, and *Pseudomonas aeruginosa*. In brief, after inoculation of single colony on the plate for culturing at 37°C overnight, DNA extraction, VBNC induction, as well as serial dilution were further performed based on the bacterial suspension.

### VBNC State Induction

The culture of *P. acidilactici* in the log phase was used to induce into the VBNC state under the freezing condition (−20°C). The changing on the culturable cell number was used as an index to investigate the culturability until the culturable number was below 1 cell/ml; the cells can be regarded as those that entered into the VBNC state (Wang et al., 2011; Miao et al., 2018). The VBNC cells were finally determined by Live/Dead BacLight bacterial viability kit (Thermo Fisher Scientific, USA) (Liu et al., 2018a) in combination with fluorescence microscopy. The growth curves, including total cell numbers, culturable cell numbers, and viable cell numbers, were determined and further analyzed as described previously.

### CPA Detection Method Design

The CPA primer was designed to distinguish *P. acidilactici* based on *pheS* gene, which is a housekeeping gene. Primers used in this study were designed using Primer Premier 5 (Table 1) (Singh et al., 2018; Xu et al., 2018). The crude DNA from *P. acidilactici* and other bacterial strains used as templates for CPA amplification was prepared from overnight culture in MRS broth. DNA was extracted using whole genomic DNA extraction kit (Dongsheng Biotech, Guangzhou) according to the manufacturer's instructions. The DNA concentration and quality were measured using a Nano Drop 2000 (Thermo Fisher Scientific Inc., Waltham, MA, USA). The qualified DNA samples were stored at −20°C until further use (Wen et al., 2020).

**Table 1:** Primer sequences of CPA for detection.

	**Primers**	**Sequence (5^**′**^-3^**′**^)**
*pheS*	4s	GGAGCCATCCGTTGAAGT
	5a	GTCAGGACCAAGGCCAAA
	2a/1s	AATGTTCGCTGCCCGTAG CGGTTGGATTGAAGTGTT
	2a	AATGTTCGCTGCCCGTAG
	3a	ACATTGGGATGCACCATGCC

### Development of CPA Detection Assay

Cross priming amplification reaction was carried out in a total 26 μl reaction mixture containing 20 mM Tris-HCl, 10 mM (NH_4_)_2_SO_4_, 10 mM KCl, 8 mM MgSO_4_, 0.1% Tween 20, 0.7 M betaine (Sigma, USA), 1.4 mM of dNTP mix, 8 U of *Bst* DNA polymerase large fragment (NEB, USA), 1.0 μM primer of 2a/1s, 0.5 μM (each) primer of 2a and 3a, 0.6 μM (each) primer of 4s and 5a, 1 μl DNA template, and 1 μl mixed chromogenic agent, and the total reaction mixture was made up of 26 μl with nuclease free water (Xu et al., 2012a). The mixed chromogenic agent was composed of 0.13 mM calcein and 15.6 mM MnCl_2_·4H_2_O. Mixture without DNA template was used as negative control (Xu et al., 2012a). The reaction mixtures are maintained at 63°C for 1 h followed by 80°C for 2 min. The amplified products were detected by electrophoresis on 1.5% agarose gel with ethidium bromide staining.

### Evaluation of CPA Methodology

The specificity of CPA assay was evaluated by amplifying genomic DNA extracted from *P. acidilactici* and 19 non-target bacteria (Table 2). Nuclease free water was added instead of DNA template as blank control. The CPA reaction was conducted under the corresponding conditions mentioned above.

**Table 2:** Reference strains and results of PCR and CPA assays.

	**PCR**	**CPA**
Reference strain no	*pheS*	*pheS*
*Pediococcus acidilactici*	+	+
*Salmonella enterica* ATCC29629	–	–
*Salmonella enterica* ATCC14028	–	–
*Listeria monocytogenes* ATCC19114	–	–
*Listeria monocytogenes* ATCC19116	–	–
*Escherichia coli* O157:H7 ATCC43895	–	–
*Escherichia coli* O157:H7 E019	–	–
*Escherichia coli* O157:H7 E020	–	–
*Escherichia coli* O157:H7 E043	–	–
*Vibrio parahaemolyticus* ATCC27969	–	–
*Vibrio parahaemolyticus* ATCC17802	–	–
*Pseudomonas aeruginosa* ATCC27853	–	–
*Pseudomonas aeruginosa* C9	–	–
*Pseudomonas aeruginosa* C40	–	–
*Staphylococcus aureus* ATCC23235	–	–
*Staphylococcus aureus* 10085	–	–
*Staphylococcus aureus* 10071	–	–
*Lactobacillus casei*	–	–
*Lactobacillus acetotolerans* BM-LA14527	–	–
*Lactobacillus plantarum* BM-LP14723	–	–

### Development of PMA-CPA for Detection of VBNC Cells

**Figure 1:**
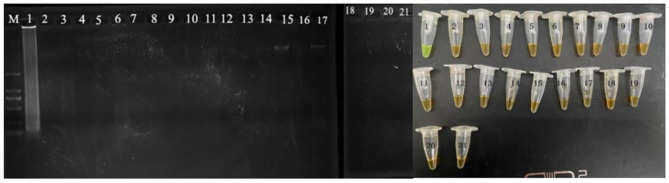
Specificity of CPA detection for different strains with *pheS* genes by 1.5% agarose gel electrophoresis and mixed chromogenic agent; M-DNA marker; lane/tube 1, *P. acidilactici* BM-PA17927; lane/tube 2–20, non-*P. acidilactici* BM-PA17927 strains of *E. coli, Salmonella enteric, Vibrio parahaemolyticus, Pseudomonas aeruginosa, Listeria monocytogenes, Staphylococcus aureus*, and *Lactobacillus casei*; lane 21, negative control.

The evaluation of the CPA assays was conducted by using serially 10-fold diluted genomic DNA and applied in food samples with 10–10^8^ cells/ml. The CPA products were detected by electrophoresis on 1.5% agarose gel with ethidium bromide staining (Xu et al., 2019). All the tests were performed in triplicate. The developed CPA assay was used to detect the VBNC cells of *P. acidilactici* combined with the PMA dying. Furthermore, the PMA-CPA was applied to detect the VBNC cells in the food samples using *pheS* gene as target. The products were analyzed by 1.5% agarose gel electrophoresis and fluorescent dye (MgCl_2_ and calcein). The ladder-like bands were observed under UV light. The color of fluorescent dye that changes reaction system from orange to green indicates positive result (Figure 1).

### Formation of VBNC State in Three Types of Artificially Contaminated Food

In this study, three major types of rice/flour products in China were selected, which were mantou, rice noodle, and Cantonese pastry. Mantou, rice noodle, and Cantonese pastry are among the top list of common food in China, with a large consumption market. The sample processing had been performed as follows. Each sample of mantou, rice noodle, and Cantonese pastry weighs 25 g, and a total of 100 μl with bacterial suspension was added to each sample. All mantou, rice noodle, and Cantonese pastry samples were further stored and subjected to CFU counting at different time points. For the first few days, CFU was performed for each day, and after 3 days, CFU was performed every 3 days.

### Application of the PMA-CPA Detection of VBNC Cells in Mantou

In order to further confirm if the established PMA-CPA methodology is capable of detection in the *P. acidilactici* VBNC state, the food samples (mantou, rice noodle, and Cantonese pastry) contaminated with *P. acidilactici* in the VBNC state was used as template. The PMA-treated bacteria suspension was centrifuged at 10,000 r/min for 5 min, and supernatant was removed. DNA was then extracted using a bacterial whole genomic DNA extraction kit (Dongsheng Biotech, Guangzhou). All samples including mantou, rice noodle, and Cantonese pastry were processed according to the standard procedure, and the PMA-CPA was performed accordingly.

## Results and Discussion

### Establishment of CPA Assay

**Figure 2:**
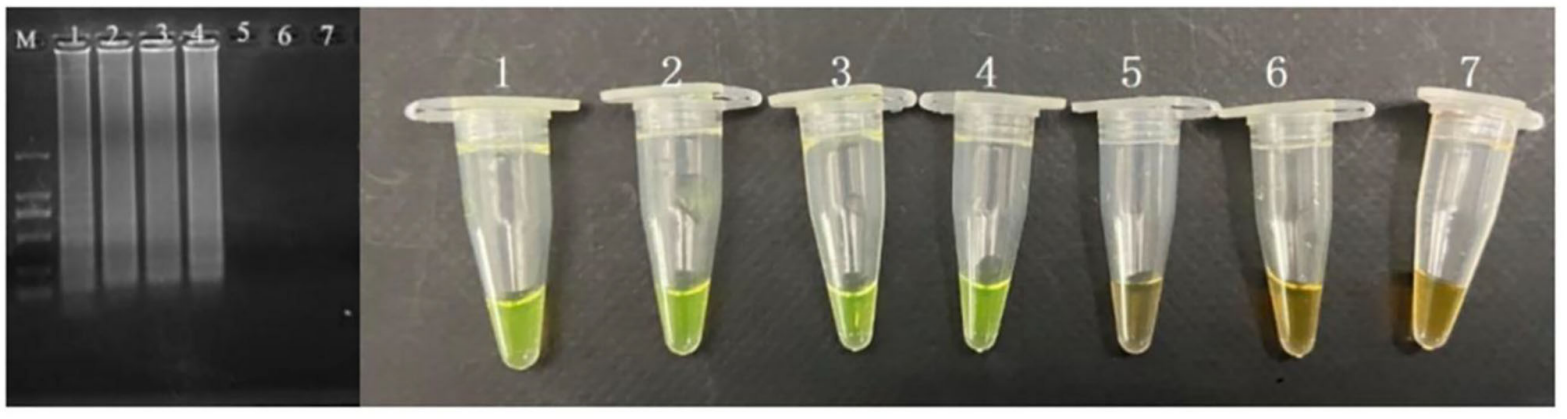
Sensitivity of the CPA assay in genomic DNA of *P. acidilactici* with *pheS* genes by 1.5% agarose gel electrophoresis and mixed chromogenic agent; M-DNA marker; 1–6 refer to 52, 5.2 ng/μl, 520, 52, 5.2 pg/μl, and 520 fg/μl; 7 refers to negative control.

A novel and simple nucleic acid isothermal amplification CPA has been developed to detect the species specific gene *pheS*. According to the results, the CPA based assay is capable of detecting *P. acidilactici* cells. According to the evaluation of the CPA assay conducted by series of DNA, the detection limit of CPA was 52 pg/μl for *pheS* gene (Figure 2).

### Applicability of CPA Assay on the Detection of *P. acidilactici*

**Figure 3:**
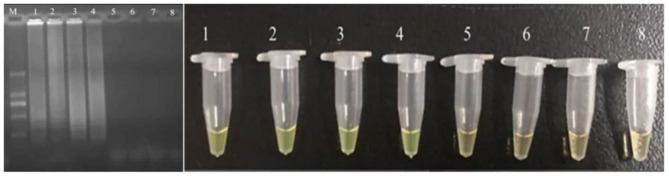
Sensitivity for *pheS* in artificially contaminated food samples. M-DNA marker; 1–7 refer to 10^7^, 10^6^, 10^5^, 10^4^, 10^3^, 10^2^, and 10 cells/ml; 8 refers to negative control.

The developed CPA assay was utilized in detection of food samples. The bacteria were inoculated into food samples with the concentration of 10–10^8^ cells/ml. The results showed that the detection limit of application was 10^4^ cells/ml (Figure 3). Compared with previously reported PCR based methodologies, the CPA assays had shown significant advantages in terms of sensitivity, specificity, rapidity, and simplicity in operation.

### Development of PMA-CPA Assay for VBNC *P. acidilactici* Cells

**Figure 4:**
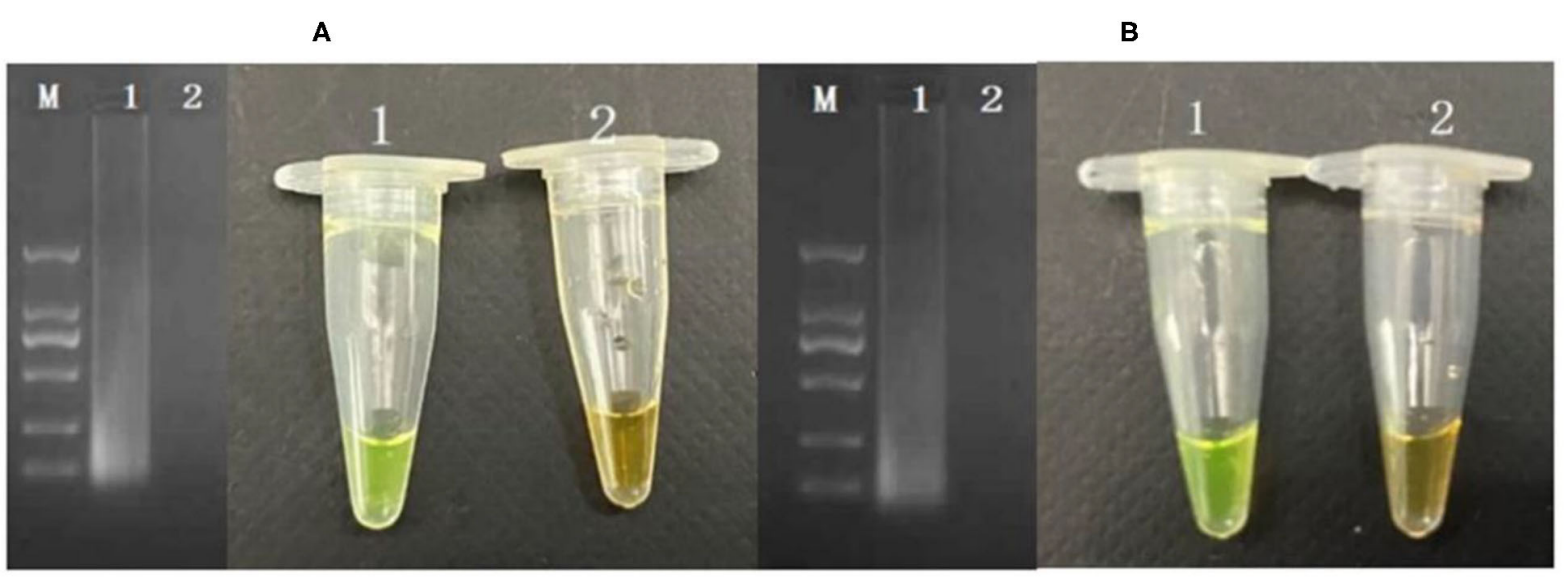
Application of detection of VBNC cells in pure cultures **(A)** and food samples **(B)**. M-DNA marker; 1: VBNC cells in pure culture; 2: dead cells in pure culture.

The cells considered may enter into the VBNC state when the culturable cell number in the induction solution is under 1 cell/ml. In order to determine whether *P. acidilactici* cells in the induction solution enter the VBNC state, the LIVE/DEAD® BacLight™ bacterial viability kit (Thermo Fisher Scientific, USA) was used (Berney et al., 2007; You et al., 2012). For these assays, 500 μl of *P. acidilactici* culture sample was obtained and centrifuged at 5,000 r/min for 15 min. Subsequently, the *P. acidilactici* cells were washed twice with PBS. The supernatant was removed, and the pellet was resuspended in 500 μl of PBS. The cell suspensions were incubated with 1.5 μl of dye mixture containing SYTO 9 and PI for 30 min at room temperature in the dark. The mixture (5 μl) was observed under a fluorescent microscope. The viable cells including normal and VBNC state showed green, and the dead cells are in red. For PMA-CPA assay, the VBNC cells were added to a 1.5 ml centrifuge tube and were thoroughly mixed and left at room temperature for 10 min. Subsequently, the centrifuge tube was placed on a crushed ice box, and light treatment was performed for 15 min at 15 cm from a 650 W halogen lamp for PMA and DNA binding. The PMA molecules remaining after the treatment are passivated. The extracted DNA was detected by CPA. The results showed that the VBNC cells can be detected successfully by PMA-CPA (Figure 4A).

### Application of PMA-CPA on Direct Detection of VBNC Cells From Food Products

The developed PMA-CPA assay as above had been further applied to detect VBNC cells of *P. acidilactici* from food samples including mantou, rice noodle, and Cantonese pastry. Firstly, formation of VBNC cells had been performed in artificially contaminated food samples including mantou, rice noodle, and Cantonese pastry. Following a rapid processing method, the samples had been further subjected to PMA-CPA detection. According to the results (Figure 4B), food samples of mantou, rice noodle, and Cantonese pastry containing *P. acidilactici* had yielded positive results. However, samples containing either none of strains (negative control) or non-target microorganisms had yielded negative results. The results demonstrated the fact that the developed method can be used in direct detection of VBNC cells of food samples (Figure 4B).

## Conclusion

As concluded, from a widely existed human probiotic bacteria and the interest for its accurate detection as an important indicator of the health status of human microbiota, including intestine and vagina, which will be significantly correlated with urinary tract infection, a major concern had been raised as how to accurately detect the probiotic commensal LAB as they are mostly hard to culture. Consequently, in this study, we had firstly developed a rapid, sensitive, and specific detection assay on *P. acidilactici* based on CPA methodology, which had obtained high sensitivity and specificity. Then, we had connected the CPA assay with PMA processing to achieve a PMA-CPA assay to directly identify the VBNC cells of *P. acidilactici*. Thirdly, the developed PMA-CPA method had been further applied for direct detection of VBNC *P. acidilactici* cells to show its robustness.

## Data Availability Statement

The raw data supporting the conclusions of this article will be made available by the authors, without undue reservation.

## Author Contributions

YG conceived the study and participated in its design and coordination. KW and YY performed the experimental work and collected the data. LC organized the database. TH wrote the manuscripts. YZ revised the manuscript. All authors contributed to manuscript revision and read and approved the submitted manuscript.

## Conflict of Interest

The authors declare that the research was conducted in the absence of any commercial or financial relationships that could be construed as a potential conflict of interest.
